# Can Influenza Epidemics Be Prevented by Voluntary Vaccination?

**DOI:** 10.1371/journal.pcbi.0030085

**Published:** 2007-05-04

**Authors:** Raffaele Vardavas, Romulus Breban, Sally Blower

**Affiliations:** Semel Institute for Neuroscience and Human Behavior, University of California Los Angeles, Los Angeles, California, United States of America; University of Guelph, Canada

## Abstract

Previous modeling studies have identified the vaccination coverage level necessary for preventing influenza epidemics, but have not shown whether this critical coverage can be reached. Here we use computational modeling to determine, for the first time, whether the critical coverage for influenza can be achieved by voluntary vaccination. We construct a novel individual-level model of human cognition and behavior; individuals are characterized by two biological attributes (memory and adaptability) that they use when making vaccination decisions. We couple this model with a population-level model of influenza that includes vaccination dynamics. The coupled models allow individual-level decisions to influence influenza epidemiology and, conversely, influenza epidemiology to influence individual-level decisions. By including the effects of adaptive decision-making within an epidemic model, we can reproduce two essential characteristics of influenza epidemiology: annual variation in epidemic severity and sporadic occurrence of severe epidemics. We suggest that individual-level adaptive decision-making may be an important (previously overlooked) causal factor in driving influenza epidemiology. We find that severe epidemics cannot be prevented unless vaccination programs offer incentives. Frequency of severe epidemics could be reduced if programs provide, as an incentive to be vaccinated, several years of free vaccines to individuals who pay for one year of vaccination. Magnitude of epidemic amelioration will be determined by the number of years of free vaccination, an individuals' adaptability in decision-making, and their memory. This type of incentive program could control epidemics if individuals are very adaptable and have long-term memories. However, incentive-based programs that provide free vaccination for families could increase the frequency of severe epidemics. We conclude that incentive-based vaccination programs are necessary to control influenza, but some may be detrimental. Surprisingly, we find that individuals' memories and flexibility in adaptive decision-making can be extremely important factors in determining the success of influenza vaccination programs. Finally, we discuss the implication of our results for controlling pandemics.

## Author Summary

Currently, a major public health concern is the next influenza pandemic; yet it remains unclear how to control such a crisis. By using novel mathematical modeling techniques, here we predict the likely impact of voluntary vaccination programs on controlling influenza epidemics and pandemics. We construct an individual-level model of human cognition and behavior that includes two important biological characteristics: memory and adaptability/flexibility. In each influenza season, each individual in the modeled population decides, using memory and adaptability/flexibility, whether to be vaccinated or not. We combine our individual-level model with an epidemic model to predict the impact of voluntary vaccination programs. We found that severe influenza epidemics cannot be prevented unless vaccination programs offer incentives. Frequency of severe epidemics could be reduced if programs provide, as an incentive to be vaccinated, several years of free vaccines to individuals who pay for one year of vaccination. However, we found that a public health intervention program that focuses on vaccinating families is likely to increase the frequency of severe epidemics. Most importantly, we found that individuals' memories and adaptability/flexibility in decision-making are critical factors in determining the success of influenza vaccination programs. Our results are applicable both for the control of seasonal and pandemic influenza.

## Introduction

Previously, both complex [1–3] and simple models [4–7] of influenza transmission dynamics have been analyzed to determine what proportion of the population would need to be vaccinated to prevent influenza epidemics and pandemics. However, none of these modeling studies have shown whether this critical coverage can actually be reached. Here we investigate, by modeling vaccination decisions made by individuals, whether the critical coverage can be achieved through voluntary vaccination. We construct an individual-level model of human cognition and behavior and link it to an epidemic model of influenza that includes vaccination dynamics. We assume that the decision of each individual is based upon self-interest such that s/he wishes to avoid catching influenza, preferably without having to be vaccinated. Since protective immunity against influenza lasts less than one year [8], individuals must decide every year whether or not to participate in a voluntary vaccination program. Individuals who get vaccinated protect themselves from infection, but if they do not get vaccinated they may still avoid infection if sufficient numbers of their peers get vaccinated (i.e., through *herd immunity*). This poses a yearly dilemma for the self-interested individual of whether vaccination is necessary. We model each individual's strategy for making yearly vaccination decisions as an adaptive process of trial and error. We track both individual-level decisions and population-level variables (yearly vaccine coverage level and influenza prevalence; where prevalence is defined as the proportion of the population that is infected). We use our model to address the following question: can influenza epidemics be prevented by voluntary vaccination?

Our individual-level adaptive decision-making model is inspired by Minority Game methodology. A Minority Game models how noncommunicating selfish individuals reach a collective behavior with respect to a common dilemma under adaptation of each one's expectations. In the past decade, Minority Games [9] have been used to model inductive reasoning systems [10] and financial markets [11]. Our constructed model consists of a population of *N* individuals acting in their own self-interest who do not communicate their vaccination decisions to each other. Every year, these individuals independently decide whether or not to get vaccinated against influenza using a risk-free, highly effective vaccine [12]. We assumed that the vaccine presents no real risk and that individuals do not perceive any risk from vaccination. Individuals in the model are characterized by two biological attributes (adaptability and memory) that they use when making vaccination decisions. Individuals can adapt their vaccination behavior for the current season on the basis of their memories of the consequences of their past vaccination decisions: i.e., they use cognition to make decisions. We couple our individual-level model of adaptive decision-making with a model of influenza vaccination dynamics. Our coupled models show the effect of individual-level vaccination decisions on influenza epidemiology and, conversely, the effect of influenza epidemiology on individual-level vaccination decisions. We first use our model to assess whether vaccination programs without incentives could achieve the critical coverage levels necessary to control influenza epidemics. We then assess the potential epidemiological impact of two public heath programs that use incentives to encourage vaccination.

There are two major classes of incentive-based public health programs that can be investigated with our coupled models. The first class uses incentives to correlate vaccination decisions for the same individual over many influenza seasons. The second class uses incentives to correlate vaccination decisions amongst individuals in the population in one influenza season. Many additional incentive-based vaccination programs can be formulated by combining the defining characteristics of these two classes. The first public health program that we investigate is an example of the first class of incentive-based programs. This program offers free vaccination for *y* number of years to an individual who pays for vaccination in the first year. We assume that the individual gets vaccinated each year during the *y* years of free vaccination, but that s/he also evaluates the necessity of vaccination every year. At the end of *y* years, each individual in the program then uses their evaluations to decide whether or not to re-enroll in the program. If they choose to re-enroll, they pay for vaccination that season (i.e., season *y* + 1) and receive free vaccinations for a further *y* years. The second public health program that we analyze is an example of the second class of incentive-based programs. This program vaccinates a family for free if the head of the family pays for her/his own vaccination. We assume that the head of the family decides every year whether to re-enroll in the program depending upon how many of her/his family members were infected in the previous season.

## Results

We found that influenza epidemics could not be prevented in most seasons if vaccination was voluntary and no incentives were offered (Figure 1A). This result was a consequence of individuals making vaccination decisions each year on the basis of their past experiences. When epidemics occurred, some individuals became infected; this increased the probability that they would get vaccinated in the next influenza season. Thus, the vaccination coverage gradually approached the critical value necessary for prevention (Figure 1A). Eventually, the coverage slightly exceeded the critical coverage level due to the stochastic nature of the individual-level adaptive decision-making process. At this point, an influenza epidemic did not occur; notably, this happened rarely (approximately once every 35 years; see Figure 1A). In the following season, many individuals decided that they did not need to get vaccinated, as an epidemic had not occurred in the previous season; thus vaccination coverage abruptly decreased and a severe epidemic ensued (Figure 1A). The vaccination coverage then repeated a similar cyclic dynamic (Figure 1A). If the initial vaccination coverage was larger than the critical coverage, the coverage dropped to a level below the critical coverage within a few years (unpublished data); vaccination coverage then followed the same cyclic dynamic as shown in Figure 1A. Since vaccination coverage determines the severity of an influenza epidemic, our results (as shown in Figure 1A) revealed that cyclic dynamics of influenza epidemics could simply be caused by individual-level adaptive decision-making.

**Figure 1:**
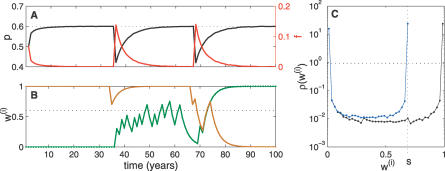
Results of the Model without Incentives The vaccination coverage dynamic has a memory parameter *s* = 0.7, an adaptability parameter ε = 1, a critical vaccination coverage level *p* *_c_* = 0.6 (dashed line), and a probability *q*(0) = 0.8 of getting infected if no one participates in the voluntary vaccination program. (A) Dynamics of yearly coverage (*p*) for a population of *N* = 10^5^ individuals (black data), and the corresponding dynamics of the prevalence (red data). The dynamics of the yearly coverage is approximately cyclic: as *p* approaches *p_c_* from below, it eventually fluctuates above *p_c_* and then abruptly drops below *p_c_*. (B) The probability that individual *i* decides to be vaccinated in season *n* is *w* ^(*i*)^ *_n_*. The figure shows *w* ^(*i*)^ *_n_* versus time for two individuals in the population. In contrast to the simple dynamics of the coverage, individuals go through complex vaccination decision behavior. (C) Normalized distributions *ρ*(*w* ^(*i)*^) versus *w* ^(*i*)^ *_n_* for a population with *N* = 10^7^ for improved accuracy. The distribution when the coverage fluctuates above *p_c_* is shown by the black data, and the distribution in the successive year when the coverage abruptly drops below *p_c_* is shown by the blue data. Individuals tend to strongly segregate into two groups. The individuals in one group are highly unlikely to get vaccinated the next season. The black data show that the individuals in the other group are highly likely to get vaccinated (i.e., *w* = 1). The blue data show that the individuals in the second group are less likely to get vaccinated than previously (i.e., given that no epidemic occurred in the previous season, *w* = *s*).

The dynamics of each individual's probability of getting vaccinated each season is more complex than coverage and prevalence dynamics (Figure 1B). Figure 1C shows the distribution containing each of the *N* individuals' probability of getting vaccinated in one season; two distributions are shown. The first distribution (black data) is obtained from a season when an epidemic does not occur. In this season, the *N* individuals segregate into two groups as has been shown for other inductive reasoning games [13]. Individuals in one group are very likely to get vaccinated whilst individuals in the other group are unlikely to get vaccinated; few are undecided. This segregated distribution results over the course of the years when the coverage is close to but below the critical vaccination coverage. During these years, both vaccination and nonvaccination behaviors are reinforcing with the small exception of a few nonvaccinating individuals who get infected. These infected individuals then begin to get vaccinated and thus increase the coverage closer toward the critical vaccination coverage. The second distribution (blue data in Figure 1) is obtained in successive seasons when severe epidemics occur. In these seasons, the distribution of the vaccination probabilities remains segregated into two groups. However, individuals who were very likely to get vaccinated previously have decreased their vaccination probability (Figure 1C), causing severe epidemics. The distribution shown by the blue data in Figure 1 slowly tended towards the distribution shown by the black data as epidemics decreased in severity. When the critical coverage level is exceeded, the distribution repeats a similar cyclic dynamic. This cyclic dynamic occurred in a homogenous population where every individual had the same memory parameter *s* and adaptability parameter *ɛ*. We found that similar cyclic dynamics occur in heterogeneous populations where memory and adaptability are normally distributed, but bounded between 0 and 1 (unpublished data). We note that, using a population-level model with a deductive reasoning game, Reluga et al. [14] have also recently shown that cyclic dynamics in vaccine coverage can occur due to heterogeneity in risk perception.

Many individuals are likely to enroll in incentive-based vaccination programs in response to a major epidemic. However, the epidemiological impact of these programs can be complex. We analyzed the potential impact of a commitment-incentive program that offers free vaccination for *y* years if the individual pays for vaccination in the first year (Figure 2). A three-year program (red data) caused substantially less severe, but more frequent, epidemics than a program without incentives (black data) (Figure 2A). In contrast, a fifteen-year program (green data) caused more frequent severe epidemics than a program without incentives. Our contrasting results are a consequence of the relationship between the length of the commitment to the program and the time scale of the memory parameter (*s; s* = 0.7 determines a half life of 1.9 years). Programs that require only a short-term commitment (e.g., *y* = 3) have a high turnover of participants and a time scale comparable to that of the memory parameter. Participants who leave this program become reinfected and therefore quickly re-enroll in the program; this process results in only small frequent epidemics. Programs that require long-term commitment (e.g., *y* = 15) have a relatively low turnover of participants and a time scale much longer than that of the memory parameter. Long-term commitment programs prevent epidemics for many years. Thus, at the end of the commitment many individuals do not re-enroll in the program because an epidemic has not occurred for many years. Therefore, vaccination coverage drops and a severe epidemic occurs; severe epidemics occur approximately every fifteen years if *y* = 15.

**Figure 2:**
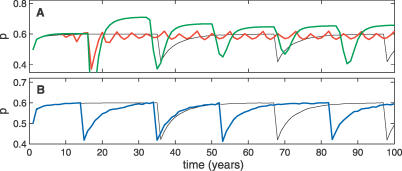
Vaccine Coverage for Different Public Health Programs. The dynamics of the vaccination coverage *p* is calculated for *N* = 10^5^ individuals using a memory parameter *s* = 0.7, an adaptability parameter ε = 1, a critical vaccination coverage level *p_c_* = 0.6, and a probability *q*(0) = 0.8 of getting infected when *p* = 0. (A) Individuals who pay for one vaccination are then given *y* = 3 (red data) and *y* = 15 (green data) free years of vaccination; the vaccine coverage when individuals are given no incentive to get vaccinated (i.e., no free vaccine) is shown by the black data for comparison. (B) The head of the family makes the decision as to whether or not their family gets vaccinated. The vaccine coverage when the family size is eight (*C* = 8) is shown by the blue data; the vaccine coverage when each individual makes voluntary vaccination decisions independently (rather than as a family) is shown by the black data for comparison. Similar results were obtained for family sizes of two and four.

To systematically assess the effect of memory, adaptability, and length of commitment on the success of vaccination programs, we conducted an uncertainty analysis for: (i) programs without incentives, (ii) short-term commitment (e.g., *y* = 3) programs, and (iii) long-term commitment (e.g., *y* = 15) programs (see Figure 3). We found that the magnitude of epidemic amelioration is determined by the length of commitment to the program, the individuals' adaptability, and their memory. When individuals are very adaptable and have long-term memories, commitment-incentive programs can be very effective in controlling influenza epidemics.

**Figure 3:**
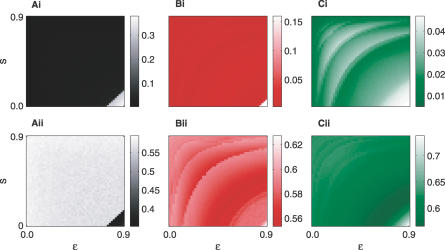
The Structure of the Average Prevalence Level (**i** Panels) and the Average Coverage Level (**ii** Panels) in the Parameter Space *ɛ* − *s* (A) Individuals receive no incentives (i.e., no free vaccines)—i.e., *y* = 0 (gray data). (B) Individuals pay for one vaccine and are then given *y* = 3 years of vaccination (red data). (C) Individuals pay for one vaccine and are then given *y* = 15 years of vaccination (green data).

As well as a commitment-incentive vaccination program, we also investigated the potential epidemiological impact of a family-incentive program. This program vaccinates a family for free if the head of the family pays for her/his own vaccination. Vaccination coverage dynamics for the family-incentive program appeared fairly similar to the coverage dynamics for the program that does not provide incentives (Figure 2B). However, surprisingly, the family-incentive program increased the frequency of severe epidemics. This result was found because epidemic severity and frequency are a function of the number of individuals who independently decide whether or not to get vaccinated. In the vaccination program without incentives, each member of the population is a decision-maker and decides independently whether to get vaccinated or not. In the family-incentive program, only one member of each family is allowed to make the decision. Therefore, the family-incentive program reduces the number of independent decision-makers from the total number of individuals to the total number of families. Stochastic variation in the coverage (and hence frequency of severe epidemics) increases as the number of independent decision-makers decreases. Thus, the family-incentive program increased the frequency of severe epidemics.

## Discussion

The critical vaccination coverage level necessary to eradicate influenza epidemics and pandemics has been calculated by analyzing influenza transmission models [1–7]. However, none of these studies have shown whether it is actually possible to reach the critical coverage level. By coupling a novel individual-level model of human cognition and behavior with an epidemic model, we have determined, for the first time, that this critical level is unlikely to be reached if vaccination is voluntary and no incentives are offered; for mathematical justification see [15]. Our modeling has shown that incentive-based vaccination programs are necessary to control influenza epidemics, but that some of these programs may be detrimental. Hence, incentive-based programs need to be carefully evaluated before they are implemented. Surprisingly, we have found that the epidemiological impact of influenza vaccination programs will depend upon the biological characteristics of individuals as well as the specific incentives that are offered.

Influenza evolution and dynamics are driven by genetic changes that can alter strain transmissibility and/or virulence; therefore, influenza epidemics can show seasonal variation in severity. The severity of an epidemic can be defined in terms of the basic reproduction number (R_0_); where R_0_ represents the average number of secondary cases caused by one infectious case at the beginning of an epidemic. Changes in strain transmissibility and/or virulence may lead to an increase (or a decrease) in the value of R_0_; thus, the value of R_0_ may show seasonal variation. However, in our analyses we used a constant value of R_0_, because we wanted to isolate the impact of individual-level vaccination decisions on influenza dynamics. It is notable that even with a constant R_0_, by including individual-level adaptive decision-making, our modeling was able to reproduce two essential characteristics of influenza dynamics and evolution: (i) annual variation in epidemic severity, and (ii) sporadic occurrence of severe epidemics. Therefore, our results suggest that individual-level adaptive decision-making may be an important (previously overlooked) causal factor in driving influenza epidemiology.

A pandemic influenza strain will not necessarily have a substantially higher R_0_ than an interpandemic (i.e., seasonal) strain. In our analyses of interpandemic strains, we used an R_0_ value of 2.5 (which requires a critical vaccination coverage of 0.6); a similar value for R_0_ has been quoted and attributed to pandemic strains [5,16]. Specifically, in Mills et al. [5], they calculate that the R_0_ values for the 1918 pandemic strains were between 2 and 3. However, it is possible that a pandemic strain may have a substantially higher R_0_ than an interpandemic strain. Therefore, we also investigated the impact of the sporadic introduction of a pandemic strain with an R_0_ = 10; this pandemic strain has a critical vaccination coverage level of 0.9. Apart from an occasional substantial increase in coverage in the year after a pandemic, the qualitative behavior of the coverage dynamics are similar to the dynamics for interpandemic influenza (unpublished data). The epidemiological impact of both the commitment-incentive and the family-incentive program on pandemics are also similar to the impact observed previously for interpandemic influenza. Therefore, we conclude that our results can be used to understand, and to predict, the potential impact of vaccination programs for controlling pandemic, as well as interpandemic, influenza.

Two previous studies [17,18], based upon a game-theoretic approach using voluntary vaccination programs (without incentives), have shown that it would only be possible to eradicate a vaccine-preventable disease if a risk-free vaccine was used. In contrast, we found that influenza epidemics are unlikely to be prevented by using voluntary vaccination with a risk-free vaccine. The reason that our results are in direct contrast to the previous two theoretical studies are that different pathogens are investigated. The earlier study by Bauch et al*.* concentrated on smallpox [18], and in the second study Bauch and Earn [17] analyzed childhood diseases. For both smallpox and childhood diseases, it is necessary to be vaccinated only once [17,18]; thus, Bauch and colleagues did not model memory nor individual adaptability. In contrast to these earlier studies, we have modeled influenza, which needs a yearly vaccination. We have assumed that individuals make their decisions both on the outcome of their own previous vaccination decisions, as well as on the basis of the previous seasons' level of herd immunity. Therefore, our model includes greater biological complexity than the previous models, as, when modeling annual influenza vaccination decisions, it is necessary to model a memory effect and to incorporate the possibility of changing behavior (i.e., adaptability). Hence we found contrasting results to the previous two studies [17,18].

The purpose of our analyses was to evaluate the role of memory and adaptation on vaccination decision-making, and also the impact of vaccination decisions on influenza epidemiology. We have presented results for a homogenous population in the memory and adaptability parameters. Similar qualitative dynamics were found for the case where the population was heterogeneous in both memory and adaptability. Many other factors may also influence individuals in making their vaccination decisions [19]. However, memory and adaptation are principle biological attributes of individuals; consequently, including them in models of recurring voluntary vaccination is essential. Our model describes a large population of individuals. We account only for epidemics and we do not consider outbreaks; outbreaks become decreasingly important as the population size *N* increases. The two central assumptions of our model are that individuals act in their own self-interest and do not communicate their vaccination decisions to each other. If these assumptions are not met, then other outcomes are possible: for example, the public may choose to continue vaccinating even if vaccination does not appear worthwhile for them. This type of behavior may be able to prevent influenza epidemics occurring. However, we stress that even a population of individuals acting in the interest of their own families would not be able to prevent influenza epidemics. Although we do not model the impact of treatment in controlling influenza epidemics, the effects of treatment can be implicitly accounted for in our individual-level model by decreasing the critical vaccination coverage level. We also do not model the economics of vaccination programs. Such analyses could be done using our model in order to assess the most cost-effective vaccination program.

In the United States, demand for influenza vaccines is generally met and no major shortages occur. In recent years, vaccination coverage (based upon voluntary vaccination) has steadily increased [20,21]. One of the national health objectives of the US is to further increase the coverage [20,21]; currently, the coverage is below the Healthy People 2010 objectives [20]. Here we have shown computationally, for the first time to the best of our knowledge, that it is unlikely that influenza epidemics will be prevented if vaccination is voluntary and no incentives are offered. We have found that incentive-based vaccination programs will be necessary for controlling influenza. We have also shown that these programs can have surprising effects and sometimes may make epidemics worse. We recommend that public health vaccination programs should be carefully evaluated before they are implemented. By modeling human cognition and behavior, we have shown that the impact of vaccination programs will depend upon both the biological characteristics of individuals as well as the specific incentives that are offered. Surprisingly, we found that individuals' memories and their flexibility in adaptive decision-making can be extremely important factors in determining the success of influenza vaccination programs.

## Materials and Methods

### Individual-level model of cognition and adaptive decision-making.

In every influenza season, each individual decides whether or not to get vaccinated, independently from each other. We assume that vaccination completely protects an individual from infection. The probability that individual *i* chooses to be vaccinated in season *n* is *w* ^(*i*)^ *_n_*. Individual *i* is assigned a vaccination experience variable *V* ^(*i*)^ *_n_*, the value of which changes every year and depends upon whether or not: the individual chose to be vaccinated, they became infected, and an epidemic occurred in the previous season (Figure 4A). *V* ^(*i*)^ *_n_* increases each time the individual perceives that there was, or would have been, a benefit to vaccination because (a) the individual got vaccinated and there was an epidemic, or (b) the individual did not get vaccinated and then became infected (Figure 4A). We model the effect of memory by using a parameter s to discount the previous seasons' vaccination outcome with respect to the outcome of the present season (0 < *s* < 1) [22]. Specifically, *V* ^(*i*)^ *_n+_*_1_ = *sV* ^(*i*)^ *_n_* + 1 if individual *i* believes s/he did, or would have, benefited from vaccination in season *n*. Otherwise, if individual *i* believes that vaccination was unnecessary in season *n* (regardless of whether s/he got vaccinated or not), *V* ^(*i*)^ *_n+_*_1_ = *sV* ^(*i*)^ *_n_*.

**Figure 4:**
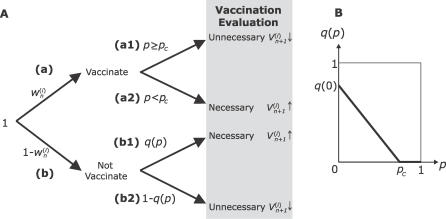
Diagrammatic Description of the Adaptive Decision-Making Model (A) Diagram illustrating the evaluation tree. An individual who decides to get vaccinated (branch (a)) will base their decision on whether there was an influenza epidemic that season. If the coverage *p* was equal to or greater than the critical coverage *p_c_* (i.e., *p* ≥ *p_c_*) (branch (a1)), they will conclude that their decision to get vaccinated that season was unnecessary to avoid infection. Otherwise, if the coverage was lower than the critical coverage (i.e., *p* < *p_c_*) (branch (a2)), they will conclude that their decision was beneficial for avoiding infection. An individual who decides not to get vaccinated that season (branch (b)) will base their decision on whether they were infected. If they did get infected (branch (b1)) they will conclude that their decision to not get vaccinated was detrimental and that vaccination would have been necessary for avoiding infection. Instead, if by chance they avoided infection (branch (b2)), they will conclude that vaccination was unnecessary. (B) The probability of getting infected with influenza *q*(*p*) versus the vaccination coverage *p*.

We normalize *V* ^(*i*)^ *_n_*_+1_ by (*s^n^* ^+1^ − 1)/(*s* − 1) because this factor is the maximum possible value for *V* ^(*i*)^ *_n_*_+1_ if individual *i* would have benefited from vaccination in all *n* influenza seasons. The domain for the *V* ^(*i*)^ *_n_* is 0 ≤ *V* ^(*i*)^ < 1/(1 − s). Note that if individual *i* has perfect memory (i.e., *s* = 1), the vaccination experience variable represents the total number of years that this individual would have benefited from being vaccinated divided by the total number of years that vaccination was available. We assume that individuals are adaptable in their decision-making as to whether to be vaccinated or not, and we use a parameter *ɛ* to describe an individuals' adaptability based upon their past experiences with vaccination (0 < *ɛ* < 1). Thus, the probability that an individual chooses to get vaccinated in the next influenza season is given by *w* ^(*i*)^ *_n_*_ +1_ = (1 − ɛ) *w* ^(*i*)^ *_n_* + *ɛ V* ^(*i*)^ *_n_*_+1_/[(*s^n^* ^+1^ − 1)/(*s* − 1)]. This expression shows that memory s and adaptability *ɛ* are not interchangeable parameters. An individual may have perfect memory of their vaccination experiences, characterized by *s* = 1, but may not use this memory if they have a small adaptability parameter *ɛ*. In our model, the probability of an unvaccinated individual acquiring influenza *q*(*p*) decreases linearly as the coverage *p* increases; Figure 4B. This function is a good approximation of the relationship found for the Susceptible–Infected–Recovered (SIR) model as well as the Susceptible–Exposed–Infectious–Recovered (SEIR) model that could be used as within-season population-level models; see below. When the vaccination coverage is greater than or equal to the critical vaccination coverage (i.e., *p* ≥ *p_c_*) the probability of an unvaccinated individual getting infected is defined to be zero.

At the end of each season, every individual evaluates their vaccination decisions based upon whether vaccination had been necessary to avoid infection. They then modify their probability to get vaccinated the next season to *w* ^(*i*)^ *_n_*_+1_. Figure 4A shows a diagram of the evaluation tree. Individuals start their first season with no prior experience in decision-making as to whether to be vaccinated or not. The initial condition assigns a random vaccination probability for the first season to every individual. Specifically, *V* ^(*i*)^_0_ = 0 and *w* ^(*i*)^_0_ is a uniformly random variable between 0 and 1. Our initial conditions were chosen to reflect the fact that the initial public awareness of the benefits of the voluntary vaccination would not be high enough to prevent an epidemic, while at the individual-level the likelihood of vaccination could vary considerably.

Our findings reported in Figure 1 are robust. Using the methodology presented in [15], we found that there exists a considerable region in the parameter space for which our model yields coverage dynamics similar to the results in Figure 1. The region is given by *ɛ* − *s* − 1 < *ɛ*(1 − *s*) [1 − *q*(0)(1 − *p_c_*)/*p_c_*] < *ɛ* − *s* + 1 and 0 < *s*(1 + *ɛ* − *s*) + *ɛ*(1 − *ɛ*) (1 − *s*)(1 − 2*s*). Furthermore, for this parameter region, there exists only one attracting state for the dynamics of the coverage. Thus, our results are independent of the initial conditions.

We investigated the potential epidemiological impact of two public health programs that we defined as a voluntary vaccination program coupled with an incentive. The first public health program that we investigated would offer free vaccination for *y* number of years if the individual paid for vaccination in the first year. We assume that during the *y* years of free vaccination the individual would continue to get vaccinated each year, but would also evaluate the necessity of influenza vaccination. At the end of their *y* years of free vaccination, every individual in the program then uses all their evaluations to decide whether to pay for vaccination that season (i.e., season *y* + 1) and further receive free vaccinations for a further *y* years. To model this public health program, the changes that are needed in the model are very few. Namely, if an individual gets vaccinated in year *n,* then *w* ^(*i*)^ *_n_*_+*r*_ = 1, with 0 < *r* < *y* + 1; thus s/he will also get vaccinated for the next consecutive *y* years (when the vaccine is provided for free).

We also investigated the potential epidemiological impact of a public health program that would vaccinate a family for free if the head of the family paid for her/his own vaccination. This is different from the model without incentives and the one with the first public health program, as in those analyses we assumed that individuals independently decide whether or not to get vaccinated. We assume that the head of the family would make her/his choice on the basis of protecting her/his family against influenza in that particular season that free vaccination is offered. S/he would then modify her/his probability of getting vaccinated in the next season depending upon how many family members became infected. To model this vaccination program we considered a population of *N* individuals who are grouped into families with *C* members. The head of the family *j* updates her/his *V* ^(*j*)^ *_n_* value (where *j* labels the family, *j* = 1...*N*/*C*) in the following way: (a) *V* ^(*i*)^ *_n_*_+1_ = *sV* ^*(i)*^_*n*_ + *C* if the head of the family had decided to have her/his family vaccinated and there was an epidemic that season; (b) *V* ^(*i*)^ *_n_*_+1_ = *sV* ^(*i*)^ *_n_* if there was no epidemic that season, regardless of whether or not the family was vaccinated against influenza; (c) *V* ^(*i*)^ *_n_*_+1_ = *sV* ^(*i*)^ *_n_* + *k,* if *k* members of the family were infected in a season where the head of the family had decided not to get her/his family vaccinated and there was an epidemic. We normalized the value of *V* ^(*i*)^ *_n_*_+1_ by a factor of *C*(*s^n^* ^+1^ − 1)/(*s* − 1) that represents the maximum possible value of *V* ^(*i*)^ *_n_*_+1_ over *n* seasons. Therefore, the vaccination probabilities are updated as follows *w* ^(*i*)^ *_n_*_+1_ = (1 − *ɛ*) *w* ^(*i*)^ *_n_* + *ɛV* ^(*i*)^ *_n_*_+1_/[*C*(*s^n^* ^+1^ − 1)/(*s* − 1)].

### Within-season epidemic model with vaccination.

To calculate the probability of getting infected with influenza *q* given a specified vaccination coverage *p* during one influenza season, we make the following assumptions that are compatible with the vaccination model presented in the main text: a) we ignore the inflow and outflow of individuals in the study population during a season (i.e., we ignore vital dynamics); b) individuals may get vaccinated against influenza only at the beginning of the influenza season; c) the vaccine is risk-free and offers perfect protection against infection; d) individuals who get infected and then recover remain immune to infection until the end of the season.

As a result of the above assumptions, we choose to model the epidemic transmission during one season using a SIR model, without vital dynamics, that includes vaccination at the beginning of each influenza season.

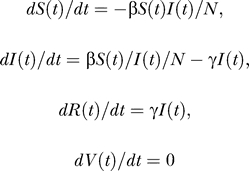
where *S*(*t*), *I*(*t*), *R*(*t*), and *V*(*t*) represent the number of susceptible, infected, recovered, and vaccinated individuals, respectively. The total number of individuals *N* = *S*(*t*) + *I*(*t*) + *R*(*t*) + *V*(*t*) is constant. *β* represents the transmissibility in the mass-action term, and *γ* represents the recovery rate. The initial conditions for the equations are as follows. A fraction *p* of the population gets vaccinated against influenza leaving only (1 − *p*)*N* susceptible individuals. Thus, at the start of the influenza season, *S*(0) = (1 − *p*) *N* − 1, *I*(0) = 1, *R*(0) = 0, and *V*(0) = *pN*.

The probability of getting infected during an influenza season *q*(*p*) is given by,

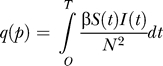
where *T* represents the duration of the influenza season. In Figure 5 we show an illustrative graph of *q*(*p*). We note that the featured dependence is approximately piecewise linear. The discontinuity in derivative occurs at *p* = 1 − 1/*N* − *γ*/*β* ≡ *π_c_* which for large *N* becomes *π_c_* ≈ 1 − *γ*/*β*.

**Figure 5:**
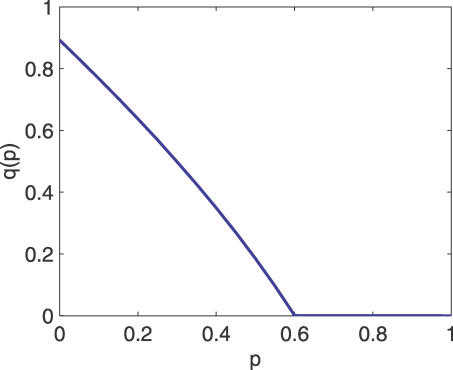
The Probability of Getting Infected *q*(*p*) versus the Vaccination Coverage *p* for the SIR Model with No Vital Dynamics The parameters are *N* = 10^5^, *β* = 5/6 day^−1^, *γ* = 1/3 day^−1^, and *T* = 200 days.

We note that a SEIR model could also be used to model influenza transmission [23]. Using an SEIR model we obtained a dependency of *q* with *p* which is similar to that presented in Figure 5 (unpublished data). Therefore, Figure 4B can be used to qualitatively model this dependency for both SIR and SEIR transmission models.

## Abbreviations

SIR: Susceptible–Infectious–Recovered, SEIR, Susceptible–Exposed–Infectious–Recovered
